# Predictors of Mortality in Light Chain Cardiac Amyloidosis with Heart Failure

**DOI:** 10.1038/s41598-019-44912-x

**Published:** 2019-06-12

**Authors:** Usman A. Tahir, Gheorghe Doros, John S. Kim, Lawreen H. Connors, David C. Seldin, Flora Sam

**Affiliations:** 1 Whitaker Cardiovascular Institute, Boston University School of Medicine, Boston, USA; 2 Cardiovascular Section, Boston University School of Medicine, Boston, USA; 3 Evans Department of Medicine, Boston University School of Medicine and Boston Medical Center, Boston, USA; 4 Alan and Sandra Gerry Amyloid Research Laboratory in the Amyloidosis Center, Boston University School of Medicine, Boston, USA; 5 Department of Biostatistics, Boston University School of Public Health, Boston, USA

**Keywords:** Heart failure, Cardiology

## Abstract

Cardiac involvement in systemic amyloidosis (AL) occurs in ~50% of all AL patients. However once symptomatic heart failure develops, therapeutic options are limited thereby conferring a poor overall prognosis. The median survival is <6 months when AL patients are untreated for the underlying plasma cell dyscrasia. We thus sought to identify risk factors of increased mortality in *treatment*-*naïve*, AL cardiac amyloidosis with heart failure. Patients with biopsy-proven AL cardiac amyloid, who presented with heart failure and did not received prior AL treatment, were enrolled between 2004–2014, at the initial visit to the Amyloidosis Center at Boston University Medical Center. Routine laboratory tests, physical examination and echocardiography data were collected. There were 165 predominantly white (76.4%), and male (61%) patients, with a mean age of 61.6 ± 9.5 years. Median survival was 10.9 months (95% CI 6.2–14.7). By multivariate analysis increased relative wall thickness (RWT) [HR 6.70; 95% CI 2.45–18.30), older age (HR 1.04; 95% CI 1.01–1.06), higher New York Heart Association (NYHA) functional class (HR 1.50; 95% CI 1.02–2.2), log brain natriuretic peptide (BNP) levels (HR 1.45; 95% CI 1.15–1.81) and C-reactive protein (CRP) levels (HR 1.02; 95% CI 1.00–1.04) were significant predictors for increased mortality. In conclusion, in treatment-naïve, AL cardiac amyloidosis patients with heart failure symptoms who lack these high-risk features may have a better outcome. These findings might allow for better risk stratification although outcomes are still poor.

## Introduction

Systemic amyloidosis (AL) is a uncommon disease characterized by the clonal proliferation of monoclonal immunoglobulin light chains resulting in extracellular fibril formation and amyloid deposits in various organs^1^. Disruption of the structure and function of normal tissue ensues and symptoms of heart failure inevitably develop. Although targeted treatment in AL amyloidosis aims to induce hematologic remission and prolong survival, only 25–30% of patients with newly diagnosed AL amyloidosis are eligible for high dose melphalan with autologous stem cell transplantation (HDM/SCT), since symptomatic cardiac involvement, more often than not, precludes this high-risk therapeutic option. While amyloid deposition in the heart reportedly occurs in up to half of all AL amyloidosis patients^2^, once symptoms of heart failure occurs, the prognosis is dismal with a median survival of <6 months if patients remain untreated^2,3^. Thus, AL cardiac amyloidosis with heart failure is a potentially fatal disease.

Biomarkers of myocardial stress and injury, such as circulating levels of N-terminal of the prohormone brain natriuretic peptide (NT-proBNP) and Troponin T (TnT) are often used to identify patients for optimal therapy selection and risk determination during treatment for systemic amyloidosis^4,5^; these biomarkers are part of the original Mayo staging for AL amyloidosis for risk stratification^4^. Subsequently, dFLC (difference in involved versus uninvolved free light chains) was introduced in the revised Mayo staging system^6^. More recently Lilliness *et al*., revised and validated the original Mayo staging to include BNP and troponin I (TnI) cutoffs in place of NT-proBNP and TnT offering centers without access to these latter markers, the ability to stage patients with systemic amyloidosis^7^. Once patients develop AL cardiac amyloidosis, however, the clinical utility of these biomarkers is more uncertain. Nordlinger *et al*., showed that BNP levels in those with cardiac amyloidosis did not discriminate between patients who had heart failure versus those who were asymptomatic. This suggests that BNP, a measure of wall stress, is unable to differentiate elevated left ventricular (LV) filling pressures from direct cardiac toxicity due to extracellular amyloid deposits^8^. Importantly, AL cardiac amyloidosis patients *with* and *without* heart failure have markedly different survival times^2,3^. Therefore, the role of these cardiac biomarkers, in those with symptoms of heart failure and AL cardiac amyloidosis, are limited. Thus, there is a need to identify additional clinical markers for prognosis and outcomes in this subgroup of patients with AL cardiac amyloidosis. We sought to identify predictors of mortality in a cohort of *treatment naïve* patients with light chain cardiac amyloidosis and heart failure.

## Methods

### Patient data collection

Data was collected over a 10-year period. Two hundred and thirty-six consecutive patients with tissue biopsy proven light chain amyloidosis featuring cardiac involvement and available clinical, laboratory, and echocardiographic data as well as serum samples obtained at initial visit to the Amyloidosis Center were selected for study. Boston University Medical Center (BUMC) Institutional Review Board approved the study (H-29147: “Biomarkers in Cardiac Amyloid”) and all subjects gave written informed consent. All research was performed in accordance with the relevant guidelines/regulations. Patients with transthyretin (TTR)-related forms of amyloidosis were excluded. None of the patients had multiple myeloma. Clinical and laboratory evaluations, including a medical history, physical examination, blood and urine tests including complete cell count, chemistries, serum/urine immunofixation and serum free light chains, chest radiography, electrocardiography and echocardiography, were systematically performed at the first visit to the Amyloidosis Center at BUMC between March 2004 and March 2014.

All subjects had a fat pad tissue biopsy positive for Congo red staining demonstrating the presence of fibrils to confirm the diagnosis of amyloidosis. In all subjects, serum and urine immunofixation electrophoresis, serum free light chain measurements, and bone marrow examination were performed to demonstrate the presence of a plasma cell dyscrasia thus establishing the diagnosis of AL amyloidosis. Amyloid cardiac involvement was determined by the presence of one of the following criteria: low voltage on electrocardiography, elevations in cardiac biomarkers, left ventricular hypertrophy on echocardiogram (in the absence of a history of hypertension or valvular disease), intraventricular septal thickness >12 mm and/or by an endomyocardial biopsy specimen that demonstrated amyloid fibril deposits. Immunohistochemical and biochemical analyses were used to identify the amyloid fibril protein.

All patients were evaluated by a cardiologist at the Amyloidosis Center and had ACC/AHA Stage C/D heart failure. Patients were classified according to New York Heart Association (NYHA) Functional class 1 to 4. Clinical course was monitored with regular follow-up at the Amyloidosis Center or by telephone and/or by contacting referring physicians, if patients were unable to return to the Amyloidosis Center. The endpoint of followup was all cause death obtained from medical records or publicly available databases.

### Echocardiography

Two-dimensional echocardiography was performed using the GE VingMed Vivid FiVe Echocardiography System (GE Vingmed, Milwaukee, WI) with a 2.5-MHz phased-array transducer as previously described^9^. Echocardiograms were performed and analyzed in a blinded manner. Left ventricular ejection fraction (LVEF) was calculated using the modified Simpson’s rule and measurements of systolic and diastolic chamber dimensions (left ventricle end-systolic diameter [LVESD] and left ventricle end-diastolic diameter [LVEDD], respectively) and wall thickness were obtained from 2D imaging according to the recommendations of the American Society of Echocardiography^9^. The standard cube formula was utilized in order to calculate left ventricular mass^10^. Relative wall thickness (RWT) was calculated as (2 × posterior wall thickness)/LVEDD.

### Biomarker analysis

Blood samples were collected at initial visit to the Amyloidosis Center. BNP, TnI and C-reactive protein (CRP) were measured in addition to routine laboratory testing. However, TnI was only routinely measured beginning in 2008. Testing was performed in the Boston Medical Center clinical laboratory. BNP and TnI was measured by Abbott chemiluminescence. CRP was measured via a rapid Automated High Sensitivity Enzyme Immunoassay (Abbott).

### Statistical analysis

Continuous variables were expressed as mean ± standard deviation and median with interquartile ranges (IQR). Categorical variables were expressed as number of patients and percentages. Univariate analyses were performed to identify factors associated with increased mortality from demographic, clinical, laboratory, electrocardiography and echocardiographic variables including age, sex, log BNP, troponin I, dFLC, RWT, CRP, LVEF, estimated glomerular filtration rate (eGFR), QRS duration, atrial fibrillation, hemoglobin, systolic blood pressure, interventricular septal thickness (IVS), LV mass index. A *P* value ≤ 0.05 was considered statistically significant. Covariates were chosen based on statistical significance and entered into a multivariate Cox proportional hazards model. Backward elimination was then conducted with *P*‐value criteria for retention set at 0.20. In order to ensure that variables were not correlated *a priori* in the multivariate model, correlation data was determined. This established that variables were not collinear, thus maintaining confidence in the present model.

To define the predictive accuracy of the Cox regression models under considerations we use the time-dependent accuracy measures (sensitivity, specificity, and ROC concepts) proposed by Heagerty *et al*.^11^. C-statistic was used to assess the discrimination of the proposed models (revised Mayo criteria which included BNP, troponin I and dFLC). Discrimination characterizes the model’s ability to correctly classify subjects for their actual outcomes.

All analyses were conducted using SAS software version 9.4 (SAS Institute Inc.) or R version 3.5.0 (R Core Team (2018). R: A language and environment for statistical computing. R Foundation for Statistical Computing, Vienna, Austria. https://www.R-project.org/).

## Results

AL amyloidosis patients presenting between 2004–2014 who had (1) cardiac amyloidosis and (2) symptoms of heart failure were enrolled (n = 236). Among these, 71 patients were excluded because they had received prior therapy directed to the plasma cell dyscrasia prior to their initial evaluation at our center. Thus, 165 non-consecutive treatment-naïve patients with AL cardiac amyloidosis and ACC/AHA Stage C/D heart failure were included in the final analyses. Demographics with clinical characteristics are outlined in Table 1. Mean age was 61.6 ± 9.5 years. The cohort was predominantly male (61%) and white (76.4%). Hypertension and atrial fibrillation occurred in 22.4% and 24.8% of individuals, respectively. All patients had heart failure symptoms and ~50% were NYHA Functional Class 3/4. The kidney was the primary non-cardiac organ affected (36.4%) and most of patients carried the lambda light chain subtype (84.2%). The majority of the patients were on loop diuretics (81.2%). Spironolactone (22.4%) and metolazone (12%) were also prescribed. Not surprisingly, beta-blocker and angiotensin converting enzyme-inhibitor/angiotensin receptor blockers were found only in 38.7% and 21.2%, respectively, as these medications are often poorly tolerated in AL cardiac amyloidosis. The duration of heart failure symptoms was 11.3 ± 8.9 months before the initial evaluation at the Amyloidosis Center. The mean time from diagnosis to the initial visit to the Amyloidosis Center was 2.4 ± 3.4 months (median of 1.0 month, range 0.0–26.0). Cardiac biopsy was performed in 41% of patients.

**Table 1:** Clinical Characteristics, Laboratory and Echocardiographic Parameters at Initial Visit to the Amyloidosis Center.

	Value (n = 165)	Median (IQR range)	Normal values
Age (years)	61.6 ± 9.5	61 (55–69)	
Sex: Male (%)/Female (%)	100 (61%)/65 (49%)		
Race: White (%)	126 (76.4%)		
Systolic blood pressure (mmHg)	112.0 ± 18.6	108.5 (101–122)	
Diastolic blood pressure (mmHg)	73.0 ± 9.9	72 (67–79)	
Pulse rate, beats/min	84.0 ± 15.2	83 (73–94)	
Body Mass Index (kg/m^2^)	25.8 ± 4.6	25.5 (23.0–28.0)	
Hypertension (%)	37 (22.4%)		
Diabetes Mellitus (%)	10 (6.0%)		
Coronary Artery Disease (%)	29 (17.6%)		
Atrial Fibrillation (%)	41 (24.8%)		
Lambda Light Chain (%)	139 (84.2%)		
Kidney Involvement (%)	60 (36.40%)		
NYHA Class	2.7 ± 0.6	2.5 (2.0–3.0)	
NYHA Class 3/4	80 (48.50%)		
ACC/AHA Stage C/D (%)	165 (100%)		
QRS duration (ms) on EKG	99.0 ± 24.5	93 (81–108)	
***Medication Use:***			
Aldactone (%)	37 (22.4%)		
Loop Diuretic (%)	135 (81.2%)		
Metolazone (%)	20 (12%)		
Beta Blocker (%)	64 (38.8%)		
ACE-I/ARB (%)	35 (21.2%)		
Anticoagulants (%)	40 (24.2%)		
***Laboratory Data:***			
BNP (pg/mL)	980.4 ± 1035.1	654 (301–1208)	0–176
Troponin I (ng/mL)	0.4 ± 0.9	0.146 (0.067–0.333)	0–0.033
C-reactive protein (mg/L)	7.6 ± 12.2	4.0 (1.0–9.5)	0–5.0
Hemoglobin (g/dl)	13.4 ± 1.7	13.4 (12.1–14.5)	11.8–16.0
Sodium (mmol/L)	138.5 ± 4.0	139 (137–141)	135–145
MDRD eGFR (ml/min/1.73 m^2^)	67.4 ± 25.5	66 (53–82)	>60
Uric Acid (mg/dl)	7.8 ± 2.6	7.35 (5.73–9.45)	2.4–6.0
Alkaline Phosphatase (U/L)	137.3 ± 86.9	110 (82–166)	25–100
Lactate dehydrogenase (U/L)	272.2 ± 76.3	259 (223.5–315.5)	171–308
Free kappa light chains (mg/L)	113.8 ± 372.8	11.2 (8.3–21.85)	3.3–19.4
Free lambda light chains (mg/L)	390.0 ± 500.4	243.0 (69.55–487.9)	5.7–26.3
Free light chains kappa/lambda ratio	11.24 ± 42.66	0.05 (0.02–0.18)	0.26–1.65
dFLC (mg/L)	478.1 ± 558.3	291.8 (119–552.6)	
***Echocardiographic parameters:***			
Left ventricular ejection fraction (%)	49.8 ± 11.6	50 (40–60)	≥50
Left atrium (mm)	40.2 ± 6.2	40 (36–44)	
Intraventricular septum (mm)	14.9 ± 2.7	15 (13–16)	6–10
Posterior wall thickness (mm)	15.2 ± 3.0	15 (13–17)	6–10
Left ventricular end systolic diameter (mm)	30.0 ± 6.2	30.0 (26.5–34.0)	21–40
Left ventricular end diastolic diameter (mm)	40.0 ± 5.8	40 (36–44)	<57
Relative wall thickness	0.8 ± 0.2	0.74 (0.63–0.89)	0.22–0.42
Calculated LV mass (g)	240.3 ± 75.7	223.7 (191.7–271.8)	67–162
LV mass index (g/m^2^)	129.2 ± 35.4	124.2 (107.5–145.5)	50–115

Data are expressed as mean ± SD for continuous variables or numbers or percent (%) for categorical variables. IQR, interquartile; AL indicates immunoglobulin light chain amyloidosis; NYHA, New York Heart Association; ECG, electrocardiogram; MDRD eGFR, glomerular filtration rate by Modification of Diet in Renal Disease equation; BNP, brain natriuretic peptide; ACE-I, angiotensin converting enzyme-inhibitor; ARB, angiotensin receptor blocker; ACC/AHA stage, American College of Cardiology/American Heart Association Classification of Heart Failure; dFLC, difference between involved and uninvolved light chains. EKG, electrocardiogram. Anticoagulants include warfarin and non-vitamin K antagonist oral anticoagulants. Normal values for echocardiography are as reported by the American Society of Echocardiography^9^.

In the original staging, Mayo Stage III disease was defined as TnT ≥0.035 ng/mL and NT-proBNP ≥332 pg/mL^4^. NT-proBNP levels and TnT were not measured at our institution. It has been proposed and validated that a BNP level of >81 pg/mL and a TnI >0.1 ng/mL provides similar discrimination for cardiac involvement^7^ as compared to NT-proBNP ≥332 pg/mL and TnT >0.035^12,13^. Thus, using this association and where both troponin and BNP were available, 64% of patients were characterized as high-risk individuals (Mayo cardiac stage III). Cardiac troponin I and BNP levels were elevated with a mean BNP of 980.4 ± 1056.0 pg/mL (median of 654 pg/mL, range: 15.0–7,161.0) and cardiac troponin I of 0.379 ± 0.980 ng/mL (median of 0.1460 ng/mL, range: 0.0–8.92). Mean LVEF was 49.8 ± 11.6% (median of 50%, range: 15–70) with ~54% of patients presenting with LVEF <50%, i.e., heart failure with reduced ejection fraction (HFrEF).

### Predictors of mortality

Univariate analyses, expressed as hazard ratios, are presented in Table 2. The strongest risk factor for all-cause mortality was increased RWT. Others included were age, NYHA functional class, atrial fibrillation, LVEF, IVS, log BNP, dFLC and CRP. Multivariate analysis (Table 2), using a Cox proportional hazards regression model with backward elimination, was used to evaluate interactions among all the risk factors for overall mortality. Age, log BNP, NYHA Class, CRP and RWT persisted as significant risk factors. Remaining univariate risk factors entered into the model were not retained by the final multivariate Cox proportional hazards model.

**Table 2:** Univariate and multivariate analysis for risk factors of mortality in AL cardiac amyloidosis with heart failure.

	Univariate Hazard Ratio	P-Value	Multivariate Hazard Ratio	P-Value
RWT	4.40 (1.87–10.30)	<0.0001	6.70 (2.45–18.30)	<0.001
NYHA Class	2.91 (2.14–3.97)	<0.001	1.50 (1.02–2.2)	0.04
Log BNP	1.93 (1.58–2.38)	<0.001	1.45 (1.15–1.81)	<0.01
Age (years)	1.04 (1.02–1.06	<0.0001	1.04 (1.01–1.06)	<0.01
CRP	1.02 (1.00–1.03)	<0.01	1.02 (1.00–1.04)	0.01
Female Sex	0.87 (0.60–1.26)	0.47	—	—
Atrial Fibrillation	1.70 (1.14–2.52)	<0.01	—	—
Systolic Blood Pressure	0.99 (0.98–1.00)	0.11	—	—
Troponin I	1.14 (0.95–1.35)	0.16	—	—
MDRD eGFR (mL/min/1.73 m^**2**^**)**	0.99 (0.98–1.00)	0.051	—	—
LVEF (%)	0.97 (0.95–0.99)	<0.0001	—	—
IVS (mm)	1.14 (1.05–1.23)	<0.001	—	—
LV mass index (g/m^**2**^**)**	0.99 (0.99–1.00)	0.88	—	—
dFLC	1.0004 (1.0001–1.0007)	0.0008	—	—
QRS duration (ms) on EKG	1.003 (0.99–1.01)	0.44	—	—

NYHA, New York Heart Association; BNP, brain natriuretic peptide; CRP, C-reactive protein; MDRD eGFR, glomerular filtration rate by Modification of Diet in Renal Disease equation; LVEF; left ventricular ejection fraction; RWT, relative wall thickness; IVS, interventricular septum; dFLC, difference between involved and uninvolved light chains; EKG, electrocardiogram.

**Figure 1:**
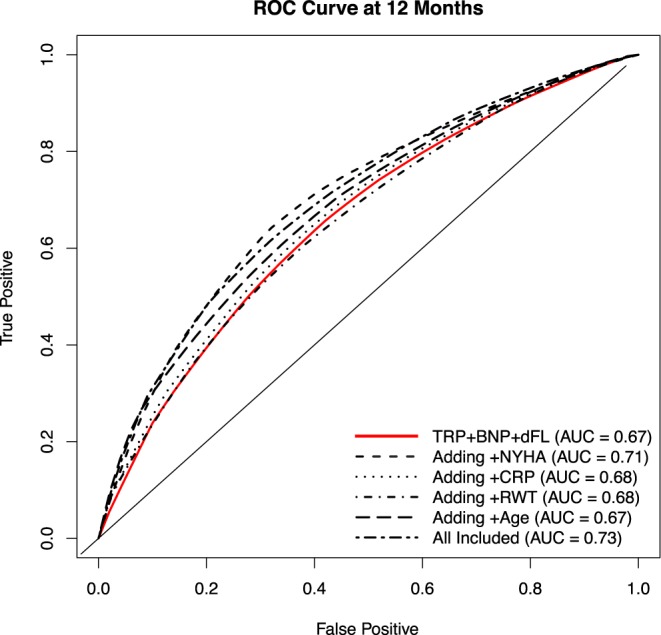
Receiver-operating characteristic (ROC) curves assessing age, NYHA class, RWT and CRP as predictors of 1-year survival in addition to standard variables (BNP, TnI and dFLC) for patients with light chain (AL) cardiac amyloidosis and heart failure.

In order to understand the contribution of these variables in addition to the revised Mayo staging system in predicting mortality, an ROC analysis was performed (Fig. 1). Each significant variable was added to the base model, which includes log BNP, TnI and dFLC. The base model had modest discriminatory ability (AUC 0.67, 95% CI 0.57–0.75). The AUC values were comparable for the addition of each significant variable to the base model: age (AUC 0.67, 95% CI 0.58–0.75), CRP (AUC 0.68, 95% CI 0.58–0.77), RWT (AUC 0.68, 95% CI 0.60–0.76), NYHA class (AUC 0.71, 95% CI (0.62–0.80). An all-inclusive model that utilized all 4 variables in addition to the base model gave the greatest power (AUC 0.73, 95% CI 0.62–0.83), in predicting 1-year mortality in AL cardiac amyloidosis with heart failure.

### Relative wall thickness

The cohort was divided into 2 groups based on RWT ≥ and < than the median of 0.74. Baseline demographics and clinical characteristics were compared between these two groups (Table 3). In the group where RWT ≥0.74, patients had worse NYHA functional class, less coronary artery disease and slightly higher hemoglobin levels. Troponin I levels were also insignificantly higher and there was no difference in renal function as measured by MDRD eGFR as compared to RWT <0.74. BNP levels and kidney involvement in AL cardiac amyloidosis did not differ between RWT ≥0.74 and RWT <0.74.

**Table 3:** Clinical and Laboratory Characteristics above and below the median for Relative Wall Thickness (RWT).

	RWT ≥0.74	RWT <0.74	P values
Age (years)	62 ± 10.1	61 ± 9.9	0.474
Sex: Male (%) Female (%)	47 (59.5%) 32 (40.5%)	50 (64%) 28 (35.9%)	0.552
Race: White (%)	55 (72.4%)	64 (83.1%)	0.081
Systolic blood pressure (mmHg)	113.4 ± 21.86	110.6 ± 14.47	0.339
Diastolic blood pressure (mmHg)	73.7 ± 10.22	71.9 ± 9.0	0.260
Pulse rate, beats/min	84.9 ± 16.9	81.9 ± 13.8	0.238
Body mass index (kg/m^2^)	25.4 ± 3.9	26.0 ± 5.5	0.411
Hypertension (%)	15 (19%)	18 (23.1%)	0.529
Diabetes mellitus (%)	3 (3.8%)	5 (6.4%)	0.697
Coronary artery disease (%)	9 (11.4%)	20 (26%)	0.019*
Atrial fibrillation (%)	20 (25.3%)	20 (26%)	0.925
Lambda light chain (%)	63 (80.8%)	70 (89.7%)	0.114
Kidney involvement (%)	33 (41.8)	26 (33.8%)	0.303
NYHA class	3 (1–4)^†^	2.5 (2–3.5)^†^	0.059
NYHA class 3/4	44 (55.7%)	32 (41%)	0.066
QRS duration (ms) on EKG	97.4 ± 25.86	101.9 ± 25.15	0.275
***Medication Use:***			
Aldactone (%)	19 (24.1%)	16 (20.5%)	0.594
Loop diuretic (%)	67 (84.8%)	63 (81.8%)	0.616
Thiazide diuretic (%)	8 (10.1%)	11 (14.3%)	0.427
Beta blocker (%)	29 (36.7%)	34 (43.6%)	0.379
ACE-I/ARB (%)	15 (19%)	15 (19.2%)	0.969
Anticoagulants (%)	15 (19%)	25 (32.1%)	0.060
***Laboratory Data:***			
BNP (pg/mL)	739 (372–1368)^†^	556 (241–972)^†^	0.107
Troponin I (ng/mL)	0.57 ± 1.39	0.22 ± 0.33	0.089
Creatinine kinase (U/L)	135 ± 20.3	103 ± 9	0.161
C-reactive protein (mg/L)	6.53 ± 7.43	8.68 ± 14.15	0.244
Hemoglobin (g/dl)	13.61 ± 1.72	13.04 ± 1.65	0.035*
Sodium (mmol/L)	138.5 ± 3.9	138.7 ± 3.6	0.729
MDRD eGFR (ml/min/1.73 m^2^)	67.9 ± 24.8	66.9 ± 21.7	0.792
Uric acid (mg/dl)	7.99 ± 2.49	7.56 ± 2.52	0.297
Alkaline phosphatase (U/L)	131.5 ± 77.1	142.7 ± 95.4	0.431
Free kappa light chains (mg/L)	163.7 ± 478.3	63.45 ± 228.7	0.096
Free lambda light chains (mg/L)	401.3 ± 475.7	378.2 ± 522.5	0.773
Free light chains kappa/lambda ratio	15.51 ± 52.79	7.42 ± 31.72	0.247
dFLC (mg/L)	536.8 ± 581.4	418.5 ± 535.0	0.188

Data are expressed as mean ± SD or median (range) for continuous variables or numbers (%) for categorical variables. ^†^Median (IQR range). AL indicates immunoglobulin light chain amyloidosis; NYHA, New York Heart Association; EKG, electrocardiogram; MDRD eGFR, glomerular filtration rate by Modification of Diet in Renal Disease equation; BNP, brain natriuretic peptide; ACE-I, angiotensin converting enzyme-inhibitor; ARB, angiotensin receptor blocker; dFLC, difference between involved and uninvolved light chains; EKG, electrocardiogram. Anticoagulants include warfarin and non-vitamin K antagonist oral anticoagulants.

As expected IVS (16.2 ± 2.5 *vs*. 13.6 ± 1.6 mm), posterior wall thickness (17.1 ± 2.8 *vs*. 13.3 ± 1.9 mm) and LV mass index (136.5 ± 36.8 vs. 121.8 ± 32.6 g/m^2^) were all significantly increased in RWT ≥0.74 vs. RWT <0.74. Despite the increase in LV mass and wall thickness, LV chamber size was significantly smaller in RWT ≥0.74 than RWT <0.74 (LVESD: 27.9 ± 5.14 *vs*. 32.1 ± 6.6 mm and LVEDD: 36.5 ± 4.5 *vs*. 43.5 ± 4.8 mm). LVEF was no different between RWT ≥0.74 and RWT <0.74 (49.2 ± 12.0 *vs*. 50.7 ± 11.7%).

**Figure 2:**
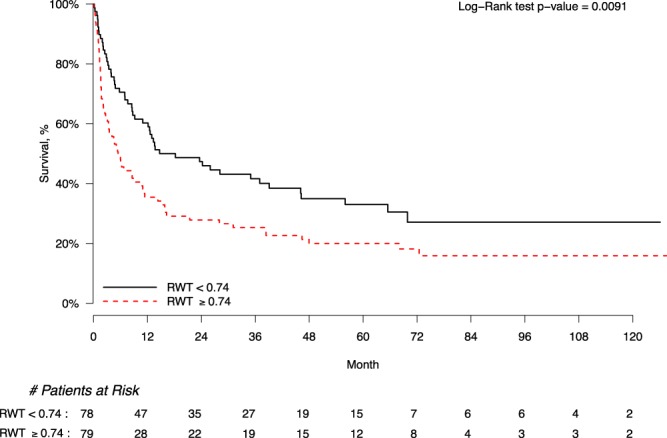
Kaplan Meier Survival curve for RWT above and below the median in AL cardiac amyloidosis with heart failure. Survival was significantly worse in patients with RWT ≥0.74 than those with RWT <0.74 (P < 0.01).

The Kaplan-Meier estimate depicts a significantly higher mortality in the group with RWT ≥0.74 *vs*. RWT <0.74 over 10 years (P < 0.01; Fig. 2). The mortality rate at 1-year followup in RWT ≥0.74 was 65%. Median survival time in RWT ≥0.74 was 5.5 months (95% CI 3.1–10.5) with a mean length of followup of 22.1 months. Conversely, the mortality rate at 1-year followup in RWT <0.74 was 39.7%. With RWT <0.74, the median survival time was 16.5 months (95% CI 9.2–39.1; P < 0.01) and the average length of followup was 30.7 months.

## Discussion

Despite some improvement in outcomes^14^ and advances in novel therapies for systemic AL amyloidosis^15^, AL patients presenting with cardiac amyloidosis and symptoms of heart failure have the greatest risk of morbidity and mortality. These patients are routinely excluded^16^ from certain life-saving therapeutic options such as HDM/SCT and also from investigational clinical trials. As seen in this study, AL cardiac amyloidosis patients with symptoms of heart failure presented late and survival was poor. Therefore, it is important to identify clinical variables that could identify patients that were lower risk and thus might be more amenable to higher risk therapies and clinical studies.

RWT, age, BNP, NYHA class, and CRP were markers of mortality in this cohort. RWT is the relationship of LV mass to volume and is the ratio of LV wall thickness to chamber radius. In subjects without cardiovascular disease but with hypertension, increasing RWT is associated with increased mortality^17^. The change in LV structure has deleterious consequences including deterioration in systolic pump performance and reduction in stroke volumes^18^. LV mass and wall thickness is greater in ATTRwt cardiac amyloidosis and the cardiac hypertrophy is more often asymmetric compared to AL cardiac amyloidosis^19,20^. But RWT is much greater in AL cardiac amyloidosis^19^ because of the smaller LV cavity in this subgroup of cardiac amyloidosis. The cardiac toxicity of amyloid deposits in AL amyloidosis induces cellular oxidative stress, cardiac myocyte dysfunction, impaired cellular autophagy and accelerates cell death^21^. These may constrain the dilation of the LV that is normally seen with eccentric cardiac remodeling and hypertrophy^22^ in cardiovascular diseases.

Given a significant majority of our AL amylodosis heart failure patients were Mayo cardiac stage III, the RWT provides an important tool to further aid clinicians in determining prognosis, as patients with RWT above and below the median have significantly different mortality rates. Therefore, increased RWT in AL cardiac amyloidosis with evidence of symptoms of heart failure, in addition to being a marker to discriminate cardiac amyloid hearts from those *without* amyloid fibril deposition^23^ ; RWT is useful in identifying “at risk” subjects and provides a simple non-invasive assessment of amyloid fibril burden on LV structure derived from routine echocardiographic measurements, thus conferring its advantage over more novel echocardiographic parameters such as left ventricular strain.

CRP is released after tissue injury/inflammation and is an acute phase reactant. CRP is also an inflammatory marker for cardiovascular events and predicts outcome after myocardial infarction^24^ and *non-amyloid* heart failure^25^. Importantly, AL amyloidosis is not considered a pro-inflammatory disease, unlike AA (amyloid A) which is associated with underlying chronic infections. However, as previously reported, circulating levels of “cardiocentric” matrix metalloproteinases (responsible for extracellular matrix homeostasis)^19^ are elevated in AL cardiac amyloidosis, suggesting a possible inflammatory *milieu* in the heart in AL cardiac amyloidosis. Although the present findings suggest that CRP may be important in identifying risk in AL cardiac amyloid with symptomatic heart failure, it is not a known risk marker in AL cardiac amyloidosis and needs to be validated in other cohorts to better define the role of inflammatory responses in AL cardiac amyloidosis.

Non-specific presenting symptoms often delay the diagnosis of amyloidosis. This is likely reflected by the lengthy time lapse (~11 months) between the onset of symptoms to presentation for initial evaluation at the Amyloidosis Center. Rahman *et al*. showed a similar delay in diagnosis from the onset of symptoms (15.4 months)^5^. In the present study, this time delay likely contributed to the advanced presentation in symptomatic AL cardiac amyloidosis patients with heart failure, with very elevated BNP and troponin levels at the initial presentation to the Amyloidosis Center. The delay in diagnosis has adverse ramifications on survival and on the ability to provide appropriate and effective treatment, specifically HDM/SCT.

Amyloid fibril deposition in the heart causes a restrictive cardiomyopathy with diastolic impairment^19,21^ leading to a reduced stroke volume, while LVEF remains relatively preserved^26,27^. In addition to diastolic function abnormalities, systolic performance is often compromised, even prior to the manifestation of cardiac involvement on standard echocardiography^27^. Using strain imaging by echocardiography, which was not available for this retrospective analysis, others showed that regional myocardial impairment is evident earlier indicating compromises in systolic pump performance prior to LVEF deterioration and symptomatic heart failure^27^. Noticeably about half of our cohort had depressed systolic function, i.e., LVEF <50%. As evidenced not only by this data, but also by worse NYHA functional class and elevated cardiac biomarkers, AL cardiac amyloidosis patients presenting with symptoms of heart failure, represents a more advanced stage of presentation.

### Limitations of the study

This retrospective, single center cohort study has some limitations. *First*, the Amyloidosis Center at BUMC is a referral center for the diagnosis and treatment of systemic AL amyloidosis and thus, a referral bias is possible in the selection of patients. Patients also presented at varying stages of disease as noted by the mean duration of heart failure ~11 months and NYHA Class ranged from 2–4. Furthermore, the availability of treatment data after initial referral visit was also limited since many patients were subsequently managed by their primary hematologist/cardiologist. Insights can be gained from the analysis of data of subsequent therapy in patients who received treatment at BUMC. Following the initial visit, 68/76 (89%) of individuals received either chemotherapy alone (63%) or HDM/SCT (37%) after their index visit. Thus, although these patients presented at advanced stages of cardiac disease, they did continue to receive standard treatments. *Second*, prior studies in AL amyloidosis cohorts showed that cardiac troponin was a risk factor for mortality, a finding not replicated in our cohort. However, cardiac troponin was not part of the initial standard testing at our Amyloid Center until 2008, thus likely influencing its omission from our multi-variate predictive model. *Third*, the findings are likely center-specific and validation in other cohorts is needed. And finally, not all patients received cardiac biopsy to verify cardiac amyloidosis. However, this is in line with the more contemporary care of these patients, as more non-invasive modalities are used to diagnosis cardiac amyloid involvement^28^.

In conclusion, AL cardiac amyloidosis patients presenting with symptoms of heart failure have poor outcomes. Other than levels of NT pro-BNP and troponin and perhaps systolic blood pressure^16^, there have been limited use of clinical data to further risk stratify patients with heart failure due to AL cardiac amyloid. In the present study RWT, older age, worse/higher NYHA functional class, increasing BNP and CRP levels were risk factors for all-cause mortality, with RWT being a simple, non-invasive tool to further risk stratify this already high-risk cohort. Given the limited life expectancy in patients with AL cardiac amyloidosis who develop heart failure, further studies are warranted to determine the effectiveness of these variables in risk stratification and their ability to help guide treatment choices in this high-risk cohort.

## Data Availability

The datasets generated during and/or analysed during the current study are available from the corresponding author on reasonable request.
